# The Role of Neutrophil-to-Lymphocyte Ratio as a Predictor of Orchiectomy or Testicular Atrophy After Torsion in Children: A Multicentric Study

**DOI:** 10.3390/jpm15070310

**Published:** 2025-07-13

**Authors:** Carlos Delgado-Miguel, Javier Arredondo-Montero, Julio César Moreno-Alfonso, Isabella Garavis Montagut, María San Basilio, Irene Hernández, Noela Carrera, Leopoldo Martínez, Estíbalitz Iraola, Inmaculada Ruiz Jiménez, Pablo Aguado Roncero, Ennio Fuentes, Ricardo Díez, Francisco Hernández-Oliveros

**Affiliations:** 1 Pediatric Surgery Department, Fundación Jiménez Díaz University Hospital, 28040 Madrid, Spainrdiez@quironsalud.es (R.D.); 2 Institute for Health Research IdiPAZ, La Paz University Hospital, 28046 Madrid, Spain; 3 Pediatric Surgery Department, Complejo Asistencial Universitario de León, 24008 León, Spain; javier.montero.arredondo@gmail.com; 4 Pediatric Surgery Department, Navarra University Hospital, 31008 Pamplona, Spain; juliomoreno.md@gmail.com; 5 Research Hotbed in General Surgery and Subspecialties, El Bosque University, Bogotá 110311, Colombia; igaravis@unbosque.edu.co; 6 Pediatric Surgery Department, La Paz University Hospital, 28046 Madrid, Spain; 7 Pediatric Surgery Department, Rey Juan Carlos University Hospital, 28933 Móstoles, Spain; 8 Pediatric Surgery Department, Toledo University Hospital, 45007 Toledo, Spain; 9 Pediatric Surgery Department, Villalba University Hospital, 28400 Villalba, Spain; eiraobla@myuax.com (E.I.);; 10 Pediatric Surgery Department, Infanta Elena University Hospital, 28342 Valdemoro, Spain

**Keywords:** spermatic cord torsion, orchiectomy, testicular atrophy, biomarkers, pediatrics

## Abstract

**Introduction**: Neutrophil-to-lymphocyte ratio (NLR) is an inflammatory biomarker (hemogram-derived-ratio) related to ischemic-inflammatory diseases. Its usefulness in the diagnosis of pediatric testicular torsion (TT) has recently been reported, although its prognostic implication has not been evaluated. Our aim is to analyze the role of NLR in the evolution of TT in children, determining its potential for predicting the risk of adverse outcomes such as orchiectomy or testicular atrophy. **Methods**: We performed a retrospective multicentric case-control study in patients with clinical and ultrasound suspicion of TT, in whom surgical testicular examination was performed between 2016–2022 in seven pediatric hospitals. Patients’ outcomes were analyzed according to the intraoperative and postoperative evolution (orchiectomy/testicular atrophy or not). Demographics and clinical, ultrasound and laboratory features at admission were analyzed. Sensitivity and specificity were determined by the area under the curve (AUC) represented on the receiver operating characteristic (ROC) curves. **Results**: A total of 455 patients (median age 13.2 years; interquartile range 10.6–14.4 years) were included, in whom 87 orchiectomies (19.1%) were performed and 34 cases of testicular atrophy (7.5%) were observed during follow-up (median follow-up: 10 months). When comparing clinical, ultrasound and laboratory predictors of both events on ROC curves, NLR was the most sensitive and specific parameter for predicting orchiectomy (AUC = 0.834; *p* < 0.001), as well as testicular atrophy (AUC = 0.849; *p* < 0.001). Compared with other parameters, the designed cut-off point of NLR = 5.2 had maximum sensitivity and specificity (82.2% and 77.0%, respectively) for predicting orchiectomy or atrophy after testicular torsion. **Conclusions**: NLR may be considered the best predictor for the risk of orchiectomy or testicular atrophy following torsion in pediatric patients, helping the identification of high-risk cases. It can be useful both for obtaining more accurate preoperative information on patient prognosis and for closer follow-up of high-risk testicular atrophy patients.

## 1. Introduction

Testicular torsion (TT) is a common cause of acute scrotal pain in children and adolescents, which results in decreased blood flow to the testicle and requires urgent diagnosis and surgical treatment to avoid negative consequences [1]. Twisting of the spermatic vessels leads to tissue ischemia and testicular necrosis, requiring excision of the testis (orchiectomy) in up to one-third of these patients [2]. Although the optimal window for surgical intervention is within 6 h of presentation, recent studies suggest that testicular salvage is still possible slightly beyond this timeframe; if treated within 6 h of pain onset, 90–100% of testes can be preserved, while intervention between 6 and 12 h results in salvage rates of 20–50%, and treatment after 12 to 24 h yields a significantly reduced salvage rate of only 0–10%, depending on the degree of torsion [3].

In some cases, despite reperfusion of the testis, testicular atrophy may be observed in the medium to long term. Several studies have identified certain clinical and intraoperative predictors of testicular viability following surgical detorsion, such as the duration of symptoms before surgery and the degree of testicular twisting [4,5,6,7]. However, there are scarce studies investigating the prognostic value of laboratory parameters in TT among children, with the majority of research focusing on adult patients [8,9,10].

Neutrophil-to-lymphocyte ratio (NLR) is an inflammatory hemogram-derived-ratio related to ischemic-inflammatory diseases in children such as intussusception or ovarian torsion [11,12,13]. Its usefulness in TT diagnosis has recently been reported [14], although its prognostic implications have not been evaluated. The aim of this study is to analyze the role of NLR in the evolution of TT in children, determining its potential for predicting the risk of adverse outcomes such as orchiectomy or testicular atrophy.

## 2. Methods

### 2.1. Study Design

A retrospective multicenter case-control study was performed in patients with clinical and ultrasound suspicion of TT, in whom surgical testicular exploration was performed between January 2016 and December 2022 at seven pediatric institutions (Fundación Jiménez Díaz University Hospital, Rey Juan Carlos University Hospital, Villalba University Hospital, La Paz University Hospital, Infanta Elena University Hospital, Navarra University Hospital and Toledo University Hospital). Only patients in whom urgent surgical exploration was performed on clinical and ultrasonographic suspicion of TT were included. Patients’ outcomes were analyzed according to the intraoperative and postoperative evolution (orchiectomy/testicular atrophy or not). Newborns with prenatal or neonatal testicular torsion were excluded, as well as patients with torted undescended testis and those with incomplete or missing data. The primary outcome of the study is to analyze the role of NLR as a predictor of orchiectomy and testicular atrophy in testicular torsion in pediatric patients. The secondary outcome is to analyze and compare other clinical, ultrasound and laboratory data to determine the sensitivity and specificity for predicting these negative events after testicular torsion.

### 2.2. Data Analyzed

Demographic information (age and weight), clinical presentation, color-Doppler ultrasonography (CDUS) findings, and laboratory results upon admission were documented. Clinical features encompassed the duration of testicular pain, affected side and symptoms such as abdominal pain, vomiting, dizziness, dysuria or fever (temperature ≥37.5 °C). Physical examination included assessment of scrotal swelling, absence of the cremasteric reflex and the absence of Prehn’s sign. Ultrasound results included the absence of intratesticular blood flow and testicular volumes, as well as the identification of the whirlpool sign, which is defined as a spiral twist of the spermatic cord [15]. Testicular volumes were calculated using the ellipsoid formula: length (mm) × width (mm) × weight (mm) × 0.71 [16].

Laboratory data were gathered from blood tests conducted in the Emergency Department upon the patients’ arrival. These tests encompassed a complete blood count, including leukocyte count and absolute counts of neutrophils, lymphocytes, monocytes, basophils and eosinophils. Additionally, biochemistry parameters such as glucose, fibrinogen, ion levels and C-reactive protein (CRP) levels were measured. NLR was calculated by dividing the absolute number of neutrophils by the absolute number of lymphocytes. Platelet-to-Lymphocyte Ratio (PLR) was obtained by the ratio between the total number of platelets (×10^9^/L) and lymphocytes (×10^9^/L). SIRI, Systemic Inflammation Response Index, was obtained by the following formula: neutrophil × monocytes/lymphocytes. Systemic Immune-Inflammation Index (SII) was obtained by calculating neutrophil × platelets/lymphocytes.

Intraoperative findings included the diagnosis of TT, the direction of rotation and the degree of twisting cord (DTC), as well as subsequent testicular viability. Orchiectomy was performed in those cases of non-recoverable testicular necrosis after detorsion. Follow-up clinic notes were reviewed for the presence of testicular atrophy after orchiopexy. Testicular atrophy was defined by a new reduction in size in comparison with the contralateral testicle found on clinical examination and/or Doppler ultrasound, defined as a >50% difference in volume when compared to the contralateral testis based on obvious physical examination findings, orchidometry or sonographic measurements, depending on the clinician’s preference [17]. In most centers, a follow-up ultrasound was performed 3–6 months after torsion to assess changes in testicular volume in cases where orchidopexy was performed.

### 2.3. Ethical Aspects

The study protocol adhered to the principles outlined in the Declaration of Helsinki (2013 revision). Approval was obtained from the hospital’s institutional review board before conducting this retrospective data analysis (IRB number PI-263-23). Written informed consent was deemed unnecessary given the retrospective nature of the study, the absence of human or animal samples and the anonymous collection of analytical data, all in accordance with institutional guidelines.

### 2.4. Statistical Analysis

Data were collected using Microsoft Excel software version 2010 (Redmond, WA, USA) and analyzed with Statistical Package for the Social Sciences (SPSS, version 25.0, IBM Corp., Armonk, NY, USA). The normality of variables was assessed using Kolmogórov–Smirnov and Shapiro–Wilk tests. Continuous variables following a normal distribution were presented as mean and standard deviation (SD) and were compared using the ANOVA test for independent samples. For continuous data not adhering to a normal distribution, median and interquartile range (IQR) were utilized, and the Kruskal–Wallis test was applied for analysis. Discrete variables were shown as frequency and percentage and analyzed using the Chi-square test or Fisher’s test when applicable. Odds ratios (OR) with 95% confidence intervals were calculated. All statistical analyses were two-tailed, and significance was set at *p* < 0.05. Sensitivity and specificity for orchiectomy and testicular atrophy were evaluated using area under the curve (AUC) in receiver operating characteristic (ROC) curves. The DeLong method was used to compare these curves [18]. Optimal cut-off values for maximal diagnostic accuracy of each analytical parameter were determined using the Youden index formula: “sensitivity + specificity − 1” [19].

## 3. Results

A total of 455 patients were included (median age 13.2 years; interquartile range 11.2–15.6 years), in whom 87 orchiectomies (21.6%) were performed and 34 (9.2%) cases of testicular atrophy were observed during follow-up (median follow-up: 32 months). Table 1 shows demographic, clinical and ultrasound features of the overall patient series, as well as data on patients with non-lost testes, those in whom orchiectomy was performed and those in whom testicular atrophy occurred despite testicular fixation after torsion. Patients who underwent orchiectomy were significantly younger than the other groups (*p* = 0.018) and consequently had a lower weight. The left testicle was slightly more frequently affected in all groups. Vomiting and abdominal pain were the most commonly associated symptoms, followed by dizziness, fever and dysuria. On physical examination, scrotal swelling was the most common finding in more than half of the patients, while absence of the cremasteric reflex and a negative Prehn’s sign were found in one third of cases. There were no differences in symptoms or clinical data between the groups. However, the time from symptom onset was significantly longer in orchiectomy patients (24 h) and in those with testicular atrophy (8 h) when compared to patients with a long-term viable testicle (4 h). Ultrasonographic data revealed an absence of testicular Doppler flow more frequently observed in patients with orchiectomy or atrophy (96% and 94%, respectively) compared to 88% of patients with successful testicular evolution (*p* = 0.013). No differences were observed in the detection of the whirling sign or testicular volume. Regarding intraoperative findings, the median number of twisting degrees was higher in patients with unfavorable evolution (720 degrees) compared to 360 degrees in patients without testicular loss (<0.001).

**Figure 1:**
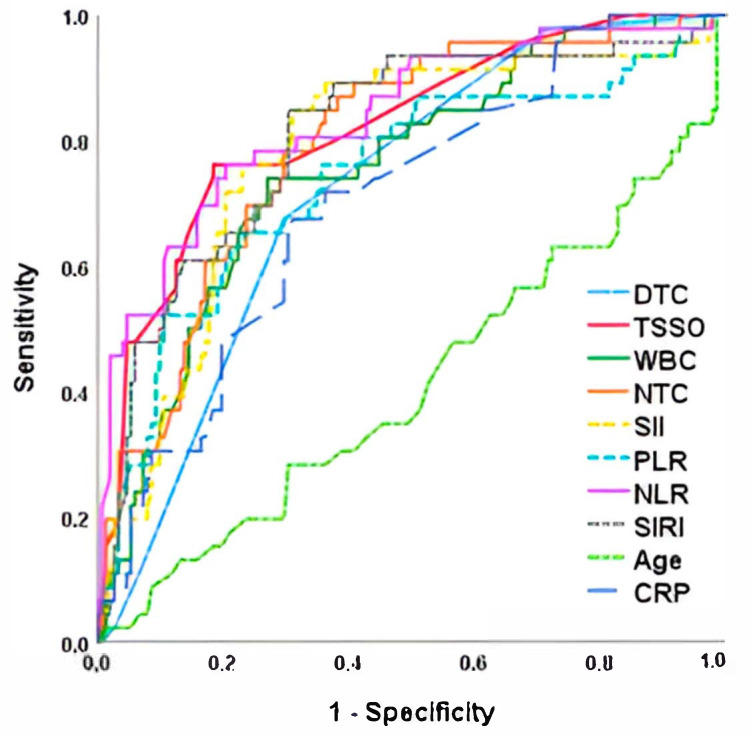
ROC curve for orchiectomy risk after testicular torsion. TSSO, time since symptom onset; DTC, degree of twisted cord; NTC, neutrophils total count; NLR, Neutrophil-to-Lymphocyte Ratio; PLR, Platelet-to-Lymphocyte Ratio; SIRI, Systemic Inflammation Response Index; SII, Systemic Immune-Inflammation Index; CRP, C-reactive protein.

Laboratory data at admission in the three groups is shown in Table 2. We observed that patients with orchiectomy or atrophy had significantly higher values of leukocytes and neutrophils, as well as inflammatory indices (NLR, PLR, SIRI and IBS) and CRP. There were no differences in biochemistry values or electrolytes. When analyzing quantitative risk factors for orchiectomy in a multivariate ROC curve analysis, we found that NLR was the parameter with the highest AUC (0.834) and the highest sensitivity and specificity for predicting orchiectomy (78.5% and 77.4%, respectively, with a cut-off point of 5.1), followed by time since symptom onset (AUC = 0.820; 76.1% sensitivity and 78.0% specificity). Figure 1 and Table 3 show the AUC values of the different parameters studied for orchiectomy prediction.

**Figure 2:**
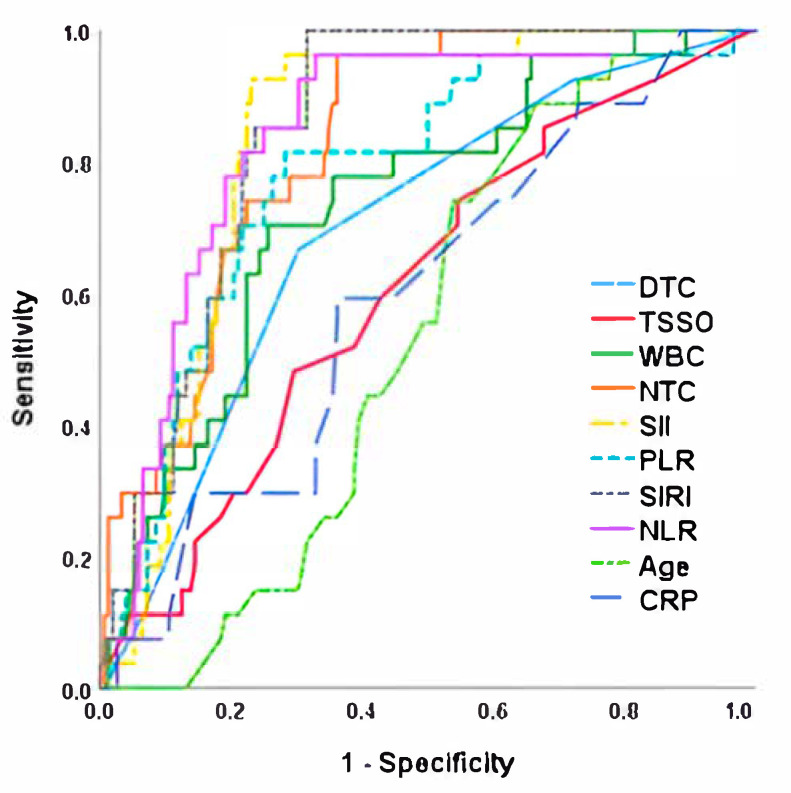
ROC curve for testicular atrophy risk after detorsion. NLR, Neutrophil-to-Lymphocyte Ratio; TSSO, time since symptom onset; SII, Systemic Immune-Inflammation Index; SIRI, Systemic Inflammation Response Index; NTC, neutrophils total count; WBC, White Blood Cells; PLR, Platelet-to-Lymphocyte Ratio; DTC, Degree of Twisted Cord; CRP, C-reactive protein.

When performing the sensitivity and specificity analysis using ROC curves for the development of testicular atrophy after detorsion, we observed that NLR was the parameter with the highest AUC (0.849), which was significantly higher than time since symptom onset, degrees of twisting cord, age at torsion and the rest of the laboratory parameters analyzed. A sensitivity and specificity of 88.9% and 77.4% was established for the cut-off point of NLR > 5.4. Detailed results, including ROC curves and the corresponding sensitivity and specificity values, as well as the determined cut-off points for the analyzed parameters, are presented in Figure 2 and Table 4.

**Figure 3:**
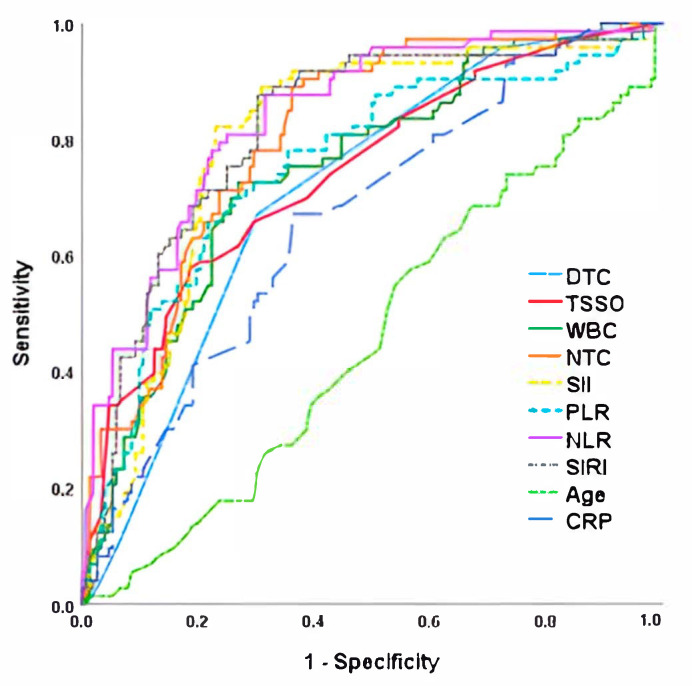
ROC curve for testicular loss (orchiectomy or testicular atrophy) after testicular torsion. NLR, Neutrophil-to-Lymphocyte Ratio; TSSO, time since symptom onset; SII, Systemic Immune-Inflammation Index; SIRI, Systemic Inflammation Response Index; NTC, neutrophils total count; WBC, White Blood Cells; PLR, Platelet-to-Lymphocyte Ratio; DTC, Degree of Twisted Cord; CRP, C-reactive protein.

Finally, when analyzing the risk of testicular loss (both orchiectomy and testicular atrophy together), we observed that the inflammatory parameters were the ones that obtained the highest AUC when compared with clinical or intraoperative data (Figure 3 and Table 5). Once again, NLR was the best predictor of unfavorable outcome (AUC = 0.838) with the highest sensitivity (82.2%) and specificity (77.0%) for the cut-off point of 5.2, followed by SII (AUC = 0.817; 78.1% sensitivity and 74% specificity for the cut-off point of 1224).

## 4. Discussion

This study analyzes for the first time the role of NLR and other inflammatory laboratory parameters as predictors of orchiectomy or testicular atrophy in boys after testicular torsion. Achieving an accurate and prompt diagnosis followed by urgent surgical intervention is imperative to prevent irreversible damage and potential loss of the testis [20,21]. Testicular torsion can manifest at any age, but it predominantly occurs after the age of 10, peaking between 12 and 16 years [22]. In our study, the median age was 13 years, and we observed a slight left-sided predominance, aligning with previous research findings [1,2]. This condition not only represents a significant cause of testicular loss in children but also carries the risk of impacting future fertility and testicular endocrine function, as well as associated psychological distress. In patients with testicular torsion, surgeons must carefully evaluate the potential complications associated with testicular preservation such as infection, malignant transformation and atrophy, against those linked to testicular removal, including trauma and psychosocial challenges during puberty. Although these risks are generally acknowledged preoperatively, the overall rate of orchiectomy following surgical detorsion remains notably high, as does the incidence of testicular atrophy after emergent orchiopexy. Consequently, identifying preoperative or intraoperative prognostic factors is crucial to optimize surgical planning and providing accurate information to patients and their parents.

In our multicenter series, the testicular loss rate of 30.8% (21.6% orchiectomy rate and 9.2% TT rate) was similar to that described by other authors [4,23], though lower than that reported by other authors with series covering a greater number of years. For instance, Tian et al. reviewed 113 cases of testicular torsion treated at their center over a ten-year period and reported an orchiectomy rate of 44.4%, which increased to 65% when cases of testicular atrophy were included [24]. Yu et al. described orchiectomy and TA rates of 26.9% and 56.6%, respectively, in 145 patients operated on over a 16-year interval [25]. These differences in atrophy rate may be due to the different definition of TA during recent years, which sometimes might be overestimated in series that include patients over more than 10 years of time.

An extended duration between the onset of pain and the diagnosis of torsion is associated with a reduced likelihood of testicular salvage [16]. In a retrospective analysis, Lian et al. reported an increased occurrence of postoperative testicular atrophy associated with delays exceeding 24 h [26]. Later, Tian et al. observed that delaying surgery beyond 12 h significantly increased testicular volume loss compared to operations performed within 12 h. Additionally, postponing surgery beyond 24 h was strongly linked to a higher risk of testicular atrophy [24]. However, the exact determination of the time interval between initial symptoms and presentation to the Emergency Department remains challenging. The low accuracy in recalling the exact onset of symptoms, combined with delays in seeking medical assistance by children and adolescents, can distort the actual timeframe from the start of testicular ischemia. Furthermore, not all cases of TT present with acute, intense scrotal pain. Some patients exhibit variable initial symptoms, including discomfort localized to the lower abdomen or inguinal region [27].

In addition to delayed surgery, the degree of torsion has also been recognized as a key prognostic factor for testicular viability during surgical intervention [4,28]. Several authors have reported a correlation between a higher degree of testicular rotation and an increased likelihood of undergoing orchidectomy compared to orchiopexy [6,29]. While lower degrees of torsion may allow partial restoration of blood flow to the testicle, leading surgeons to opt for testis-salvaging procedures in hopes of preserving function and delaying orchiectomy unless absolutely necessary, many testicles still experience substantial atrophy when monitored over the long term in clinical follow-up []. Chen et al. proposed a nomogram to estimate the likelihood of testicular salvage based on factors such as symptom duration, intratesticular blood flow, degree of spermatic cord torsion and monocyte count. However, the subjective variability of certain parameters and the limited sample size pose challenges for generalizing these findings [30]. Howe et al. identified cut-off values for testicular loss at 8.5 h and 495 degrees of torsion, offering sensitivities of 73% and 53%, respectively, with a specificity of 80% for both thresholds []. Some authors have described other clinical or ultrasound predictors of TA such as red scrotal changes or heterogeneous testis parenchyma on ultrasound, but no objective laboratory parameters such as those included in our study have been analyzed to date [25,31].

We had previously described the usefulness of NLR in the diagnosis of TT, and we have recently observed that this marker is also useful in diagnosis of ovarian torsion in the pediatric population [14]. However, its usefulness as a prognostic marker has not been explored until now. This study allows us to broaden the application of NLR not only as a diagnostic marker but also as a prognostic marker of TT. The inflammatory response triggered by testicular ischemia results in neutrophilia, driven by chemotaxis and increased release of neutrophils from the bone marrow into peripheral circulation, along with lymphopenia caused by elevated endogenous cortisol levels induced by ischemia [14]. These combined mechanisms lead to an increase in the NLR through two distinct pathways. This could account for the higher AUC observed for NLR compared to other inflammatory markers like PLR, SIRI or SII, which involve monocytes or platelets, as NLR reflects the synergistic cellular response of neutrophilia and lymphopenia more directly. This systemic inflammation observed in the laboratory study of patients with suspected TT thus provides prognostic information on the evolution and repercussions of ischemia in the short and medium term, such as orchiectomy or testicular atrophy, and makes it possible to predict them more accurately than clinical or intraoperative data.

This study included a substantial participant pool, offering significant insights into the enduring outcomes of pediatric patients with TT. In addition, it analyzes the predictive role of different clinical parameters (age at torsion, time since symptom onset, degree of twisting cord), together with laboratory data commonly used in clinical practice, all derived from the hemogram. Furthermore, it examines for the first time several inflammatory markers, including NLR, PLR, SIRI and SII, which can be easily derived from routine blood tests, and compares these markers with clinical and ultrasound findings, mimicking everyday clinical scenarios. The results highlight NLR as the most accurate and reliable indicator for predicting testicular loss either by orchiectomy or testicular atrophy in male children after testicular torsion. This parameter is more consistent than clinical observations, such as the duration of symptoms, which can be unreliable due to the gradual onset of pain in some instances. Furthermore, NLR eliminates the variability associated with radiological evaluations, such as ultrasound, which can depend heavily on the examiner’s expertise. All this facilitates the practical use of the findings on a day-to-day basis, which makes them highly applicable in clinical practice.

However, there are several limitations to consider. Firstly, the retrospective design confines the analysis to data already recorded in medical records, potentially introducing recall and selection biases. Additionally, the lack of similar studies in pediatric patients complicates direct comparison of results. Another limitation is the variability in ultrasound imaging, as it was not consistently performed by the same radiologist, which may affect the accuracy of testicular volume calculations, Doppler flow identification or whirlpool sign visualization. In addition, inflammatory markers may be elevated by other acute inflammatory processes, such as viral infections at different levels (respiratory tract, gastrointestinal tract, etc.), so it cannot be guaranteed that it is exclusively testicular ischemia, as inflammatory phenomena at other levels could coexist and contribute to the inflammatory response observed in the laboratory data. Furthermore, the study did not explore the impact of testicular atrophy on hormone production, semen quality and fertility, which added to the relatively short-term follow-up, meaning that long-term outcomes such as testicular atrophy and infertility may not be fully captured. It is possible that testicular development during puberty could reveal significant volume loss in pre-pubertal boys who experienced testicular torsion. Therefore, caution is advised when generalizing from these findings, and further prospective studies with longer follow-up periods are still needed to validate these results.

## 5. Conclusions

The neutrophil-to-lymphocyte ratio (NLR) may be considered the strongest predictor of the risk of orchiectomy or testicular atrophy following torsion in pediatric patients, presenting the highest sensitivity and specificity for both conditions. It may prove useful both for obtaining more accurate preoperative information regarding the patient’s prognosis aiding in the identification of high-risk cases and for closer monitoring of patients at elevated risk of testicular atrophy, and it could have significance in clinical practice.

## Figures and Tables

**Table 1:** Demographic, clinical and ultrasound features collected in both groups.

	Total (n = 455)	Non-Lost Testicles (n = 334)	Orchiectomy (n = 87)	Testicular Atrophy (n = 34)	*p*-Value *
**Age (years); median (IQR)**	13.2 (10.6–14.4)	13.3 (11.8–14.6)	11.9 (3.9–14.2)	13.2 (11.4–14.3)	0.018
**Weight (kg); median (IQR)**	48.5 (33.4–57.1)	50.1 (42.3–58.0)	44 (30.5–53.5)	51 (38.2–60.1)	0.002
**Testicle involved; n (%)**					0.276
**• Right**	209 (45.9)	155 (46.4)	38 (43.7)	16 (47.1)	
**• Left**	246 (54.1)	179 (53.6)	49 (56.3)	18 (52.9)	
**Associated symptoms; n (%)**					
**• Abdominal pain**	93 (20.4)	70 (21.0)	15 (17.2)	8 (23.5)	0.051
**• Vomiting**	133 (29.2)	104 (31.1)	20 (23.0)	9 (26.5)	0.184
**• Dizziness**	16 (3.5)	15 (4.5)	0	1 (2.9)	0.065
**• Disuria**	4 (0.9)	3 (0.9)	0	1 (2.9)	0.329
**• Fever**	8 (1.8)	5 (1.5)	2 (2.3)	1 (2.9)	0.079
**Clinical findings; n (%)**					
**• Scrotal swelling**	249 (54.7)	192 (57.5)	37 (42.5)	20 (58.8)	0.059
**• Absent cremasteric reflex**	175 (38.5)	130 (38.9)	33 (37.9)	13 (38.2)	0.543
**• Negative Prehn’s sign**	143 (31.4)	104 (31.1)	28 (32.1)	11 (32.4)	0.215
**Time since symptoms onset (hours); median (IQR)**	5 (3–12)	4 (2–8)	24 (10–48)	8 (6–12)	<0.001
**Ultrasound findings;**					
**• Absence of intratesticular Doppler flow; n (%)**	411 (90.3)	295 (88.3)	84 (96.6)	32 (94.1)	0.013
**• Whirlpool sign; n (%)**	129 (28.4)	91 (27.2)	26 (29.9)	12 (35.3)	0.079
**• Testicular volume (cm^3^); median (IQR)**	12 (6–19)	12 (6–18)	15 (8–20)	13 (7–19)	0.095
**Degree of twisted cord (degrees); median (IQR)**	360 (360–720)	360 (180–720)	720 (360–720)	720 (360–720)	<0.001

IQR, interquartile range; * *p* values refer to comparisons between the 3 groups (non-lost testicles, orchiectomy and testicular atrophy).

**Table 2:** Laboratory variables collected in both groups.

	Non-Lost Testicles (n = 334)	Orchiectomy (n = 87)	Testicular Atrophy (n = 34)	*p*-Value
**Leukocytes (×10^9^/L)**	9.26 (7.5–11.9)	12.1 (10.0–14.6)	13.2 (10.1–15.1)	<0.001
**Neutrophils (×10^9^/L)**	5.6 (3.6–8.3)	9.61 (8.2–12.8)	9.68 (6.60–11.71)	<0.001
**Lymphocytes (×10^9^/L)**	2.4 (1.8–3.4)	1.2 (1.12–1.64)	1.65 (1.16–2.59)	<0.001
**Monocytes (×10^9^/L)**	0.55 (0.43–0.76)	0.57 (0.39–0.74)	0.90 (0.64–1.24)	<0.001
**Platelets (×10^9^/L)**	270 (228–320)	269 (235–300)	305 (234–373)	0.052
**NLR**	2.0 (1.1–4.2)	7.5 (6.1–9.2)	6.4 (3.2–8.7)	<0.001
**PLR**	107 (81–152)	209 (164.3–277.3)	175.3 (109.8–268.6)	<0.001
**SIRI (×10^9^/L)**	1.2 (0.6–2.64)	3.9 (2.7–5.75)	4.4 (1.9–9.2)	<0.001
**SII (×10^9^/L)**	557 (326–1251)	2104 (1561–2624)	1738 (875–2876)	<0.001
**CRP (mg/L)**	0.5 (0.2–2.9)	5.5 (0.9–7.4)	2.9 (0.5–9.8)	<0.001
**Glucose (mg/dL)**	102 (91–119)	109 (101–120)	100 (84–114)	0.056
**Urea (mg/dL)**	29 (25–34)	31 (25–35)	27 (22.5–32.5)	0.095
**Creatinine (mg/dL)**	0.64 (0.5–0.76)	0.63 (0.5–0.71)	0.6 (0.4–0.74)	0.509
**Fibrinogen (mg/dL)**	338 (302–398)	358 (300–463)	365 (314–481)	0.215
**Ionogram**				
**• Na^+^**	139 (137.2–104.4)	138.4 (137–140.1)	140 (137–141)	0.145
**• K^+^**	4.0 (3.7–4.3)	4.3 (3.7–4.4)	4.2 (3.8–4.4)	0.072
**• Cl^−^**	104 (102–106)	103 (102–105.7)	102 (101–104)	0.121

NLR, Neutrophil-to-Lymphocyte Ratio; PLR, Platelet-to-Lymphocyte Ratio; SIRI, Systemic Inflammation Response Index; SII, Systemic Immune-Inflammation Index; CRP, C-reactive protein.

**Table 3:** Sensitivity and specificity analysis of orchiectomy after testicular torsion using area under the curve (AUC) and 95% confidence interval (CI95%).

	AUC (CI 95%)	Cut-Off Point	Sensitivity	Specificity	*p* Value
**NLR**	0.834 (0.763–0.905)	5.1	78.5	77.4	<0.001
**TSSO**	0.820 (0.749–0.891)	8.5	76.1	78.0	<0.001
**SII**	0.806 (0.731–0.881)	1176	78.3	70.2	<0.001
**SIRI**	0.794 (0.723–0.865)	2545	76.1	77.3	<0.001
**NTC**	0.781 (0.704–0.860)	8.1	78.3	71.2	<0.001
**WBC**	0.753 (0.675–0.832)	11.3	73.9	73.0	<0.001
**PLR**	0.741 (0.653–0.830)	137.9	71.7	68.5	0.005
**DTC**	0.720 (0.644–0.797)	2	67.4	71.2	0.012
**CRP**	0.702 (0.619–0.784)	1.5	67.4	68.6	0.057
**Age**	0.411 (0.311–0.510)	10.4	54.2	48.7	0.066

AUC, Area Under the Curve; CI, Confidence Interval; NLR, Neutrophil-to-Lymphocyte Ratio; TSSO, time since symptom onset; SII, Systemic Immune-Inflammation Index; SIRI, Systemic Inflammation Response Index; NTC, neutrophils total count; WBC, White Blood Cells; PLR, Platelet-to-Lymphocyte Ratio; DTC, Degree of Twisted Cord; CRP, C-reactive protein.

**Table 4:** Sensitivity and specificity analysis of testicular atrophy after detorsion using area under the curve (AUC) and 95% confidence interval (CI95%).

	AUC (CI 95%)	Cut-Off Point	Sensitivity	Specificity	*p* Value
**NLR**	0.849 (0.792–0.906)	5.4	88.9	77.4	<0.001
**SIRI**	0.840 (0.769–0.896)	2655	85.2	77.2	<0.001
**SII**	0.833 (0.770–0.913)	1355	85.2	75.0	<0.001
**NTC**	0.829 (0.762–0.895)	2545	77.8	71.1	<0.001
**PLR**	0.779 (0.688–0.870)	158	74.1	75.0	<0.001
**WBC**	0.733 (0.635–0.830)	11.3	73.9	73.0	<0.001
**DTC**	0.697 (0.595–0.798)	1.5	64.5	70.2	0.005
**TSSO**	0.607 (0.497–0.717)	2	61.2	58.3	0.012
**CRP**	0.593 (0.484–0.703)	1.5	60.4	57.6	0.057
**Age**	0.539 (0.448–0.630)	10.4	56.2	51.9	0.066

AUC, Area Under the Curve; CI, Confidence Interval; NLR, Neutrophil-to-Lymphocyte Ratio; TSSO, time since symptom onset; SII, Systemic Immune-Inflammation Index; SIRI, Systemic Inflammation Response Index; NTC, neutrophils total count; WBC, White Blood Cells; PLR, Platelet-to-Lymphocyte Ratio; DTC, Degree of Twisted Cord; CRP, C-reactive protein.

**Table 5:** Sensitivity and specificity analysis of testicular loss (orchiectomy and testicular atrophy) using area under the curve (AUC) and 95% confidence interval (CI95%).

	AUC (CI 95%)	Cut-Off Point	Sensitivity	Specificity	*p* Value
**NLR**	0.838 (0.784–0.893)	5.2	82.2	77.0	<0.001
**SII**	0.817 (0.757–0.877)	1224	78.1	74.0	<0.001
**NTC**	0.806 (0.749–0.864)	8.1	75.3	72.0	<0.001
**SIRI**	0.799 (0.737–0.861)	2456	74.2	72.3	<0.001
**PLR**	0.753 (0.683–0.824)	148	73.6	73.1	<0.001
**WBC**	0.745 (0.678–0.813)	11.4	72.6	73.0	0.007
**TSSO**	0.741 (0.671–0.811)	137.9	71.7	68.5	0.012
**DTC**	0.709 (0.640–0.778)	5.5	69.6	66.2	0.025
**CRP**	0.663 (0.589–0.737)	0.7	67.1	64.3	0.038
**Age**	0.458 (0.378–0.537)	11.7	53.4	47.3	0.303

AUC, Area Under the Curve; CI, Confidence Interval; NLR, Neutrophil-to-Lymphocyte Ratio; TSSO, time since symptom onset; SII, Systemic Immune-Inflammation Index; SIRI, Systemic Inflammation Response Index; NTC, neutrophils total count; WBC, White Blood Cells; PLR, Platelet-to-Lymphocyte Ratio; DTC, Degree of Twisted Cord; CRP, C-reactive protein.

## Data Availability

Data are available upon reasonable request to the authors.
